# Low frequency of CD4^+^CD25^+ ^Treg in SLE patients: a heritable trait associated with *CTLA4 *and *TGFβ *gene variants

**DOI:** 10.1186/1471-2172-10-5

**Published:** 2009-01-27

**Authors:** Marta Barreto, Ricardo C Ferreira, Lara Lourenço, Maria F Moraes-Fontes, Eugénia Santos, Miguel Alves, Cláudia Carvalho, Berta Martins, Rita Andreia, João F Viana, Carlos Vasconcelos, Luísa Mota-Vieira, Carlos Ferreira, Jocelyne Demengeot, Astrid M Vicente

**Affiliations:** 1 Instituto Gulbenkian de Ciência, Rua da Quinta Grande, 6 2780-156 Oeiras, Portugal; 2 Associação dos Doentes com Lupus, Av. Defensores de Chaves 27, 4°D 1000-110 Lisboa, Portugal; 3 Clínica Universitária de Medicina II, Hospital de Santa Maria, Avenida Professor Egas Moniz 1649-035 Lisboa, Portugal; 4 Instituto de Ciências Biomédicas Abel Salazar, Largo Prof. Abel Salazar 2, 4099-003 Porto, Portugal; 5 Instituto Nacional de Saúde Dr. Ricardo Jorge, Largo 1° de Dezembro, 4049-019 Porto, Portugal; 6 Hospital Divino Espírito Santo de Ponta Delgada, EPE, Avenida D. Manuel I 9500-370 Ponta Delgada, Azores, Portugal; 7 Instituto Nacional de Saúde Dr. Ricardo Jorge, Av. Padre Cruz, 1600-560 Lisboa, Portugal

## Abstract

**Background:**

CD4^+^CD25^+ ^regulatory T cells play an essential role in maintaining immune homeostasis and preventing autoimmunity. Therefore, defects in Treg development, maintenance or function have been associated with several human autoimmune diseases including Systemic Lupus Erythematosus (SLE), a systemic autoimmune disease characterized by loss of tolerance to nuclear components and significantly more frequent in females.

**Results:**

To investigate the involvement of Treg in SLE pathogenesis, we determined the frequency of CD4^+^CD25^+^CD45RO^+ ^T cells, which encompass the majority of Treg activity, in the PBMC of 148 SLE patients (76 patients were part of 54 families), 166 relatives and 117 controls. SLE patients and their relatives were recruited in several Portuguese hospitals and through the Portuguese Lupus Association. Control individuals were blood donors recruited from several regional blood donor centers. Treg frequency was significantly lower in SLE patients than healthy controls (z = -6.161, *P *< 0.00001) and intermediate in the relatives' group. Remarkably, this T cell subset was also lower in females, most strikingly in the control population (z = 4.121, *P *< 0.001). We further ascertained that the decreased frequency of Treg in SLE patients resulted from the specific reduction of *bona fide *FOXP3^+^CD4^+^CD25^+ ^Treg. Treg frequency was negatively correlated with SLE activity index (SLEDAI) and titers of serum anti-dsDNA antibodies. Both Treg frequency and disease activity were modulated by IVIg treatment in a documented SLE case. The segregation of Treg frequency within the SLE families was indicative of a genetic trait. Candidate gene analysis revealed that specific variants of *CTLA4 *and *TGFβ *were associated with the decreased frequency of Treg in PBMC, while *FOXP3 *gene variants were associated with affection status, but not with Treg frequency.

**Conclusion:**

SLE patients have impaired Treg production or maintenance, a trait strongly associated with SLE disease activity and autoantibody titers, and possibly resulting from the inability to convert FOXP3^+^CD25^- ^into FOXP3^+^CD25^+ ^T cells. Treg frequency is highly heritable within SLE families, with specific variants of the *CTLA4 *and *TGFβ *genes contributing to this trait, while *FOXP3 *contributes to SLE through mechanisms not involving a modulation of Treg frequency. These findings establish that the genetic components in SLE pathogenesis include genes related to Treg generation or maintenance.

## Background

Immunological tolerance is a key feature of the immune system that allows the organism to discriminate self from nonself, providing defense against foreign pathogens while preventing autoimmunity. This ability of the immune system is controlled by mechanisms of central and peripheral tolerance. Central tolerance involves deletion of self-reactive T cells in the thymus at an early stage of development [1]. However, this mechanism alone is not sufficient for preventing autoimmunity, as autoreactive T cells are also detected in healthy individuals [2]. A second mechanism involves the thymic selection of a population of regulatory T cells (Treg), which dominantly prevents both the activation and the effector function of autoreactive T cells that have eluded other mechanisms of tolerance [3]. The depletion or inactivation of Treg leads to the development of a wide range of autoimmune and inflammatory manifestations such as gastritis, oophoritis, orchitis, thyroiditis, inflammatory bowel disease and spontaneous autoimmune diabetes, in animal models [4]. In addition, reduced numbers of this cellular subset have been found in murine models of SLE [5].

T cells expressing the high affinity T Cell Receptor (TCR) for antigens expressed on the thymic epithelium [6] are selected in the thymus, originating Treg cells able to suppress both proliferation and cytokine production by effector cells in a cell-contact dependent manner. The mechanisms involved in Treg generation, maintenance or function have not been fully clarified. For instance, while *CTLA-4*^-/- ^mice develop an early lymphoproliferative disease [7] and *CTLA4 *gene expression in the thymus appears instrumental for T cell selection [8], some regulatory T cells can be generated in the absence of CTLA-4 [9]. Given its wide ranged immuno-suppressor activity, TGF-β was first thought to be a mediator of Treg activity [10], but is nowadays known to play a major role in Treg homeostasis by promoting their proliferation [11].

In humans, Treg are highly enriched in the cellular subset expressing CD4, the α chain of the IL-2 receptor (CD25) and the memory marker CD45RO [12]. Low numbers or functional defects in CD4^+^CD25^+ ^T cells have been found in patients with SLE [13,14], immune-mediated diabetes [15], and multiple sclerosis [16]. Natural CD4^+^CD25^+ ^Treg express the transcription factor FOXP3, which controls their development and function [17]. Functional defects in the *FOXP3 *gene lead to the absence of Treg production and to Immunodysregulation, Polyendocrinopathy and Enteropathy, X-linked Syndrome (IPEX) [18]. The control of immune cell numbers and effector functions in response to lymphopenia, infection and autoimmune reactivity are also under the strict control of Treg [19].

SLE is a chronic systemic autoimmune disease with unknown etiology, and thought to result from the interplay between genetic and environmental factors [20]. Multiple immune system defects have been described in patients with this condition, including B cell hyperactivity and increased antinuclear autoantibody production, defects in T cell activation and aberrant cytokine production [21,22]. To establish the involvement of Treg in SLE pathogenesis, we compared the frequency of CD4^+^CD25^+^CD45RO^+ ^T cells in SLE patients and controls and correlated it with disease activity and antinuclear autoantibody production. We also analyzed the frequency of CD4^+^FOXP3^+ ^cells in a subset of patients and investigated Treg frequency variation with disease activity, severity and development and remission of symptoms in two SLE cases. Further, we analyzed the segregation of CD4^+^CD25^+^CD45RO^+ ^frequency in SLE-affected families and tested the candidate genes *CTLA4*, *TGFβ*, *FOXP3*, *IL2 *and *CD25 *for association with Treg frequency distribution and with SLE.

## Methods

### Population sample

SLE patients and their relatives were recruited in several hospitals throughout Portugal in collaboration with the Portuguese Lupus Association. A total of 148 SLE patients (136 females and 12 males) were enrolled in the present study. 76 of the SLE patients were part of 54 families, from which 166 relatives (98 females and 68 males) were also included in the study. All patients met the revised 1997 American College of Rheumatology criteria for SLE [23], independently of their disease activity at recruitment. The SLE Disease Activity Index (SLEDAI) [24] was assessed for each patient at the time of collection (SLEDAI range: 0–41). Irreversible damage caused by the disease was calculated using the Systemic Lupus Erythematosus International Collaborating Clinics (SLICC) damage index. Information on medication from each patient was collected at recruitment. Patients and their relatives completed a 10 question screening questionnaire [25], used here as an indication of autoimmune disease manifestations in each individual (Table 1). Control individuals (N = 117, 58 females and 59 males) were blood donors recruited from several regional blood centers. The population sample characteristics and the numbers used in each of the subsequent analyses are described in Additional file 1. Approval for this study was obtained from the Portuguese Ethics Committee for Life Sciences Research, and collection of all biological samples was performed after written informed consent from all participating individuals.

**Table 1:** SLE survey questionnaire and percentage of patients and relatives that gave a positive answer to each question.

SLE survey questionnaire	Positive answers	
	% of Patients	% of Relatives
1. Have you ever had painful swollen joints for more than three months?	65.0	13.6
2. Do your fingers ever change color, become numb or uncomfortable in the cold?	63.3	26.4
3. Have you ever had mouth ulcers for more than two weeks?	40.0	18.6
4. Have you ever been told that you have low blood counts (anemia, low white cell count or low platelet count)?	71.7	15.0
5. Have you ever had an obvious or prominent rash on your cheeks for more than a month?	71.7	9.3
6. Do you develop a distinct rash in the sun (not just sunburn)?	66.7	10.0
7. Has it ever been painful to take a deep breath for more than just a few days (pleurisy)?	33.3	4.3
8. Have you ever been told that you have protein in the urine?	26.7	0.0
9. Do you find a lot of hair on your pillow on waking?	43.0	10.7
10.Have you ever had a seizure, convulsion or fit?	3.0	0.7

### Cell surface staining and flow cytometry

PBMC were isolated using Vacutainer CPT™ tubes (Becton Dickinson). For the CD4CD25CD45RO staining 1 × 10^6 ^cells were fixed in 2% PFA, washed twice in PBS 2% FCS and subsequently incubated for 20 min at 4°C with the optimal dilution of each conjugated anti-human mAb (CD4-FITC, CD25-Cy-chrome, CD31-PE and CD45RO-APC, Becton Dickinson). Cells were washed again and fluorescence intensity staining was analyzed using a FACScan flow cytometer (FACScalibur™ and CellQuest™ software, Becton Dickinson). CD4^+^CD25^+ ^T cells were defined, for all the individuals, as the population of CD4 positive T cells whose CD25 expression exceeded the level of CD25 positivity seen in the CD4 negative T cells. The CD4-FITC, CD25-APC, FOXP3-PE staining was performed according to eBioscience manufacture protocol.

### Anti-dsDNA autoantibody measurement by quantitative ELISA

Reacti-Bind™ EIA plates (Pierce) were coated overnight at 4°C with 1 mg/mL purified dsDNA (Sigma-Aldrich). The plates were blocked with a solution of 1% gelatin for 5 h at 4°C and incubated overnight at 4°C with test plasma (in triplicates) at a total protein concentration was 0.5 mg/mL. Plates were then incubated overnight at 4°C with 50 μL of alkaline phosphatase-conjugated anti-human IgG (Sigma-Aldrich) and bound IgG was revealed using 1 mg/mL *p*-nitrophenyl phosphate (Sigma-Aldrich). Absorbance at 405 nm was measured in an ELISA plate reader (BioRad). Quantification of each reactivity was obtained by fitting ODs to a standard curve present in duplicates on each plate, and consisting of 12 serial dilutions of respective positive control sera. In the readout, one unit was defined as the equivalent of the highest concentration used for the respective positive control serum, which was previously chosen to be below the saturation level. Fractions of one unit were consequently defined by the dilution factor of this standard, at which it yielded an OD equivalent to that obtained for a respective sample [26].

### Genotyping

Genomic DNA was isolated from PBMC by standard methods. The polymorphic markers tested were selected for their putative role in gene expression or function. Within the *CTLA4 *gene, we genotyped a functional SNP involving a missense A>G transition at +49 exon 1 (rs231775) and a 3'UTR microsatelite marker proposed to influence mRNA stability and turnover [27]. In the *TGFβ *gene, we genotyped a C>T transition in the promoter (rs1800469) and a G>C SNP in exon 1 (rs1800471), both known to be associated with the serum concentration of TGF-β [28,29]. Within the *FOXP3 *gene two microsatelite markers were genotyped (one in intron 0, which has been described as having a promoter/enhancer function and other in intron 5) and a -1128 G>A SNP (rs12843496) [30]. The *IL2 *gene, -384 G<T (rs2069762) and +114 G>T (rs3087209) in the promoter and in exon 1, respectively, were genotyped as previously described [30,31]. Within the *CD25 *gene, we genotyped a (CA)n repeat 10 kb upstream of the gene (D10S189) and two SNPs, rs2274037 (a A>G transition in exon 4) and rs1570538 (a C>T transition in the 3'UTR region of CD25) as previously described [32]. The D10S189 microsatelite marker was genotyped using the Applied Biosystems fluorescence based automated sequencer. Semi-automated fragment sizing was performed using GENESCAN^® ^3.1.

### mRNA isolation and quantification

Total RNA was isolated from 5 × 10^6 ^PBMC using the RNeasy Mini Kit (QIAGEN). The first strand cDNA was generated by reverse transcription with oligo dT primer (Invitrogen). The CTLA-4, TGF-β, FOXP3, IL-2 and CD25 mRNA levels were quantified with the LightCycler (Roche Molecular Biochemicals) in 33 patients and 24 controls, which were representative of the total population sample. 50 ng of first-stranded cDNA were amplified and real-time fluorimetric intensity of SYBR green I was monitored. RT-PCR reactions consisted in an initial denaturation step at 95°C for 10 minutes followed by 45 cycles of denaturation at 95°C for 15 seconds, 56°C for 5 seconds and 72°C for 23 seconds. The individual samples were standardized by the amount of HPRT RNA. This RT-PCR reaction consisted on an initial denaturation step at 95°C for 10 minutes followed by 45 cycles of denaturation at 95°C for 15 seconds, 60°C for 5 seconds and 72°C for 23 seconds. Additionally, CTLA-4 and CD25 mRNA expression levels were normalized by the proportion of CD4^+ ^cells, given that these molecules are mainly expressed by CD4^+ ^T cells.

### Statistical analysis

Nonparametric analysis of variance was performed using the Mann-Whitney U and Kruskal-Wallis tests to compare the distributions of T cell frequencies in patients and controls. Results were considered significant at the 0.05 level. Heritability estimation was performed by the SOLAR software [33]. In this test the likelihood of the sporadic model of no inheritance is compared with the likelihood of a polygenic model of inheritance. The sporadic model constitutes our null hypothesis in which it is assumed that there is not a genetic component underlying the frequencies of Treg cells. The polygenic model constitutes our testing hypothesis, where the quantitative trait is considered to be determined by a large number of genetic *loci *acting independently and/or additively. The models are then compared to find the hypothesis that is better supported by the data. Hardy-Weinberg equilibrium (HWE) was analyzed by the HWE exact test. Linkage disequilibrium (LD) between two polymorphic markers was quantified as D' using the HAPLOXT program implemented in the GOLD software [34].

## Results

### Lower frequency of CD4^+^CD25^+ ^and CD4^+^CD25^+^CD45RO^+ ^cells in SLE patients

**Figure 1:**
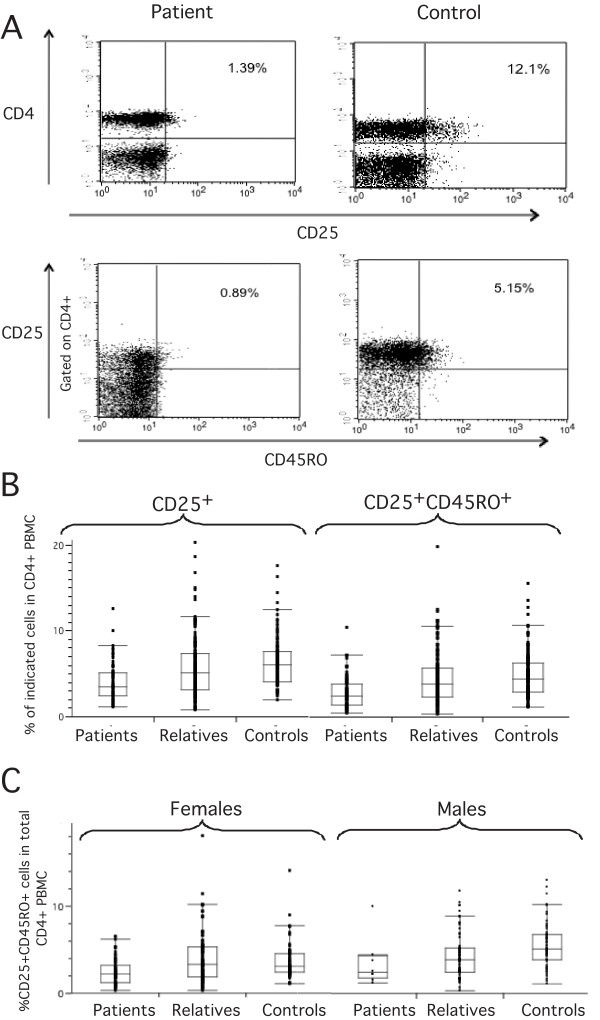
**Regulatory T cell frequency is lower in PBMC of SLE patients than in controls (A) Lymphocytes gated according to forward and side scatter (FSC/SSC) from PMBC were screened by flow cytometry for the presence of CD4^+^CD25^+ ^and CD4^+^CD25^+^CD45^+ ^T cells**. The FACS panel representing CD4^+^CD25^+^CD45RO^+ ^PBMC is gated on CD4^+ ^cells. Representative FACS stainings from an SLE patient and a healthy individual are shown, (B) box plot representing the frequency of CD4^+^CD25^+ ^and CD4^+^CD25^+^CD45RO^+ ^PBMC in patients (n = 76), relatives (n = 156) and controls (n = 193), showing that SLE patients have a significantly lower Treg frequency than healthy individuals and (C) Relative distribution of CD4^+^CD25^+^CD45^+ ^T cell frequency in CD4^+ ^T cells between male and female patients, relatives and controls, showing that females have a significantly lower frequency of CD4^+^CD25^+^CD45^+ ^T cell frequency than males.

The previous observation that a CD4^+^CD25^+ ^T cell subpopulation expressing the CD45RO activation marker exhibits 75% higher suppressive activity [35] suggests that the majority of Treg activity is restricted to CD45RO^+ ^cells. We therefore evaluated the frequency of both CD4^+^CD25^+ ^and CD4^+^CD25^+^CD45RO^+ ^T cell populations in total lymphocyte PMBC samples of SLE patients, their relatives and healthy controls and expressed it as a percentage of total CD4^+ ^cells (Figure 1A). CD4^+^CD25^+ ^and CD4^+^CD25^+^CD45RO^+ ^T cell frequencies were significantly decreased in patients compared to controls (Mann-Whitney U test, *z *= -6.339, *P *< 0.00001 and *z *= -6.161, *P *< 0.00001, respectively). CD4^+^CD25^+ ^and CD4^+^CD25^+^CD45RO^+ ^T cell distribution in relatives was intermediate between that of patients and controls (Figure 1B). As the frequency distributions of either cell population were identical, the results obtained for the CD4^+^CD25^+^CD45RO^+ ^cell subset was considered for the remaining of this study.

SLE affects primarily women and consequently only about 10% of our patients are male, while our control population is balanced in terms of gender. We therefore tested whether this gender bias was not a confounding factor in our interpretation. CD4^+^CD25^+^CD45RO^+ ^T cell frequency was significantly lower in healthy females than in males (Mann-Whitney U test: z = 4.121, *P *< 0.001). The CD4^+^CD25^+^CD45RO^+ ^T cell frequency was also lower in females than in males in the patient and relative groups, although the difference did not reach statistical significance (Figure 1C). Separate analysis within each gender group shows that the CD4^+^CD25^+^CD45RO^+ ^T cell frequency is always significantly lower in patients (Mann-Whitney U test, Males: z = -2.504, *P *= 0.012; females: z = -3.864, *P *< 0.001).

CD25 is a marker for both Treg and activated cells. To rule out the possibility that this T cell population is mainly constituted by activated cells we measured the frequency of the CD4^+^CD45RO^+ ^T cells, since CD45RO expression is associated with early activation events. No difference was found in the frequency of CD4^+^CD45RO^+ ^cells between patients and controls (Mann-Whitney U test: z = -1.724, *P *= 0.089), indicating that the CD25^+ ^population was largely a regulatory population.

**Figure 2:**
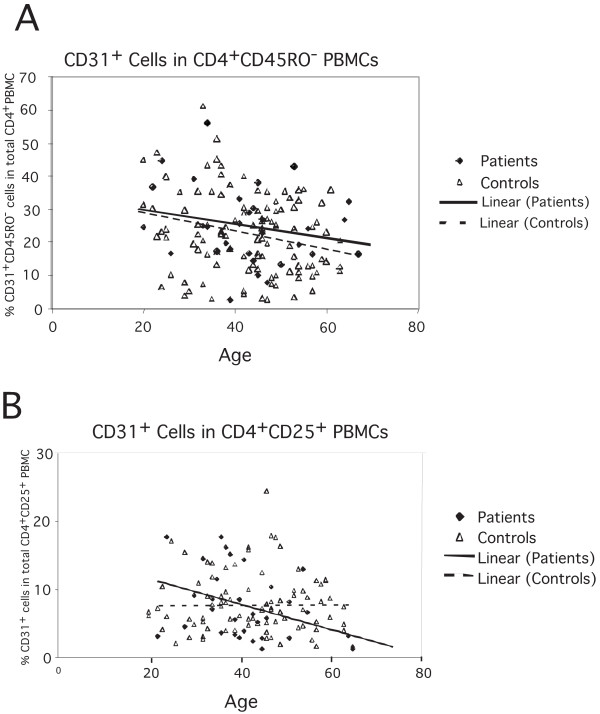
**(A) Decrease in the frequency of recent thymic emigrants with age, as indicated by the decrease of the CD45RO^-^CD31^+ ^population in total CD4^+ ^PBMC in SLE patients and controls**. (B) Decrease in the frequency of CD31^+ ^cells inside CD4^+^CD25^+ ^cells with age, as indicated by the decrease of the CD45RO^-^CD31^+ ^population in total CD4^+ ^PBMC in SLE patients and controls The linear regression curve is shown for each group.

To exclude a global thymic output defect, we evaluated the percentage of recent thymic emigrants (RTE) inside the CD4^+ ^population using the phenotypic markers CD45RO^-^CD31^+ ^[36]. As expected, CD45RO^-^CD31^+ ^cell frequency decreased with age, but there were no differences between patients and controls in absolute frequency or in the kinetics of decay (Figure 2A). Moreover, the frequency of RTE did not correlate with the percentages of CD4^+^CD25^+^CD45RO^+ ^cells (ρ = 0.10, *P *= 0.094). However, the CD31^+ ^cells inside CD4^+^CD25^+ ^cells decreased faster with age in patients than in controls (Figure 2B), suggesting a defect specifically in thymic Treg cell production in SLE patients. No significant difference was found in the frequency of CD4^+^CD45RO^-^CD31^+ ^or CD31^+ ^cells inside CD4^+^CD25^+ ^between males and females (data not shown).

### SLE patients display higher frequency of CD4^+^FOXP3^+ ^cells which are mainly CD25^-^

**Figure 3:**
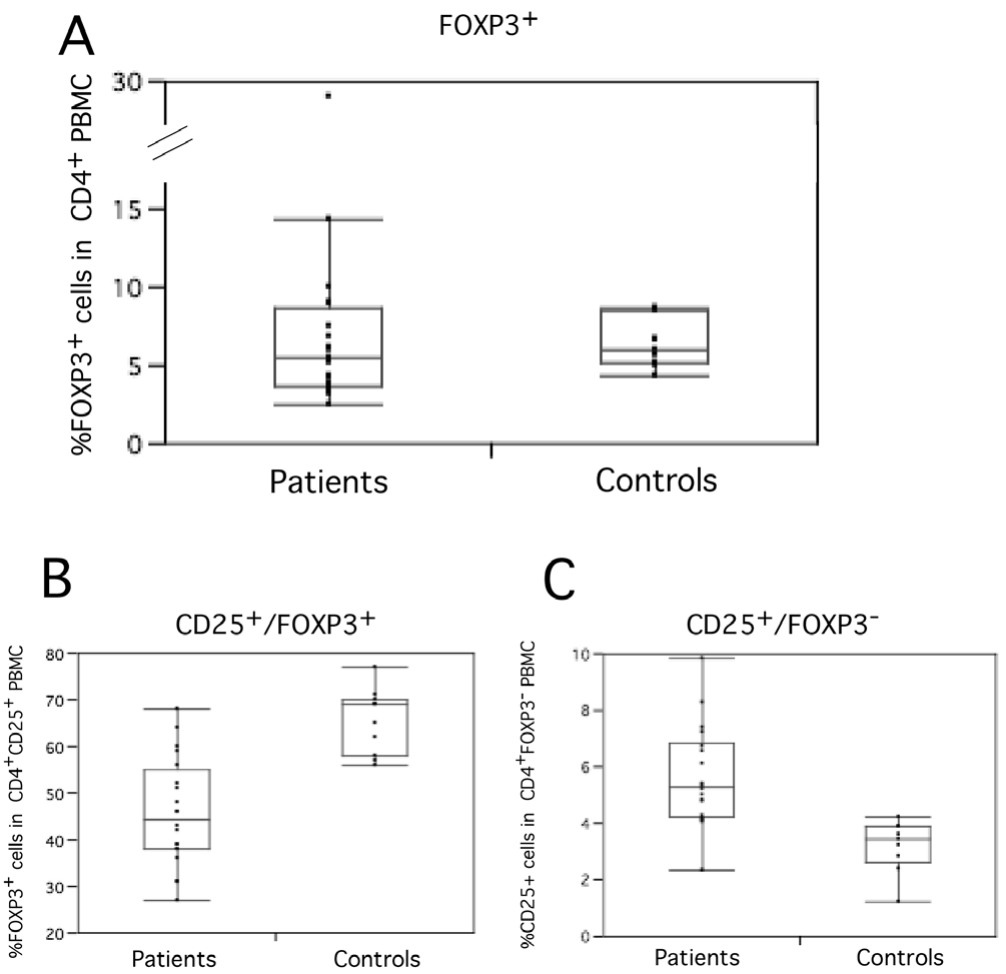
**Relative distribution of (A) FOXP3^+ ^cells in total CD4^+ ^PBMC (B) CD25^+ ^cells in total CD4^+^FOXP3^+^PBMC and (C) CD25^+ ^cells in total CD4^+^FOXP3^- ^PBMC between patients (n = 19) and controls (n = 10)**.

Given the major involvement of FOXP3 in the differentiation and function of Treg, we analyzed the frequency of FOXP3^+ ^cells in a subset of 19 patients and 10 healthy individuals. While the frequency of FOXP3^+ ^cells in CD4^+^PBMC was not significantly different between patients and controls (Figure 3A), the frequency of CD25^+ ^in the CD4^+^FOXP3^+ ^cell subset was significantly reduced in patients when compared to healthy controls (Mann-Whitney U test, *z *= 2.457, *P *= 0.014) (Figure 3B). In contrast, FOXP3^-^CD25^+ ^activated cells were significantly increased in SLE patients (Mann-Whitney U test, *z *= -2.377, *P *= 0.017) (Figure 3C). This diminished expression of CD25 in patients' FOXP3^+ ^cells indicates that the overall reduction in CD4^+^CD25^+ ^cells in SLE patients likely results specifically from a defect in the ability to convert FOXP3^+^CD25^- ^into FOXP3^+^CD25^+ ^cells.

### CD4^+^CD25^+^CD45RO^+ ^T cell frequency is negatively correlated with SLE-associated phenotypes

**Figure 4:**
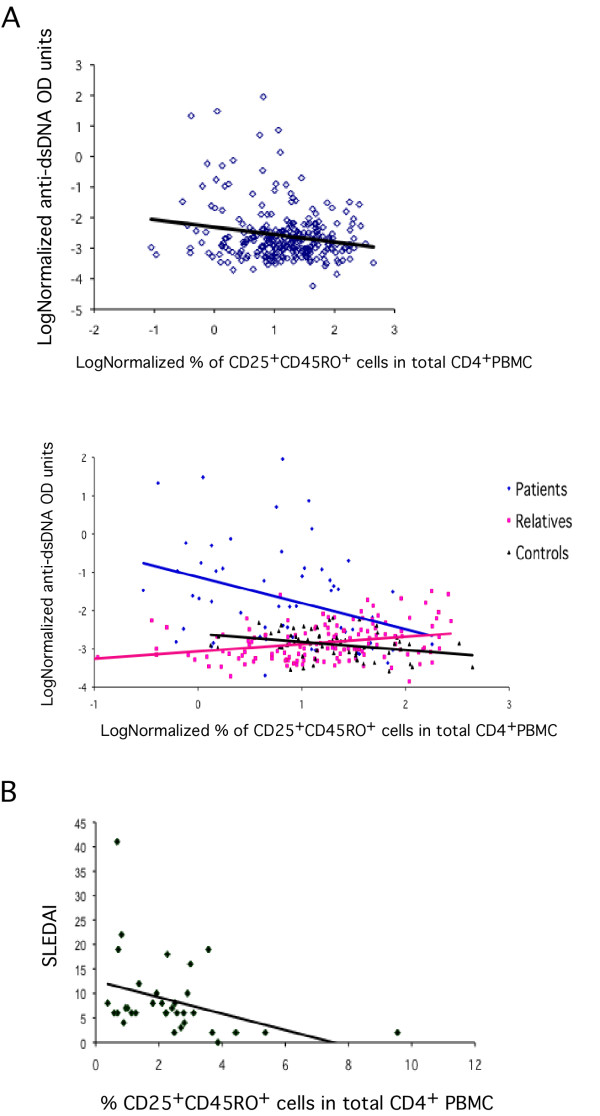
**(A) Negative correlation between CD4^+^CD25^+^CD45RO^+ ^T cell frequency and anti-DNA antibody titers in our population sample and correlations between CD4^+^CD25^+^CD45RO^+ ^T cell frequency and anti-DNA antibody titers in patients relatives and controls (B) Negative correlation between the percentage of CD25^+^CD45RO^+ ^in total CD4^+ ^PBMC cells and SLE disease activity index (SLEDAI) (n = 54)**.

Autoantibody production against dsDNA is a hallmark of SLE. We therefore analyzed the plasma immunoreactivity profiles against dsDNA from SLE patients, relatives and healthy individuals. Overall, there is a negative correlation between Treg frequency and anti-dsDNA in the total population (ρ = -0.19, *P *= 0.0026) (Figure 4A), reinforcing the notion that perturbations in the system that maintains immunologic unresponsiveness to self-constituents lead to the loss of peripheral tolerance and to SLE. This negative correlation between anti-dsDNA autoantibodies and the frequency of CD4^+^CD25^+^CD45RO^+ ^T cells was also found in our patient (ρ = -0.37, *P *= 0.005) and control groups (ρ = -0.30, *P *= 0.022) (Figure 4A). Surprisingly, there is a positive correlation between the frequency of CD4^+^CD25^+^CD45RO^+ ^T cells and anti-dsDNA antibodies in the relatives' group (ρ = 0.29, *P *= 0.0004) (Figure 4A). No difference was found between males and females.

The CD4^+^CD25^+^CD45RO^+ ^T cell frequency was inversely correlated with disease activity measured by the SLEDAI index (ρ = -0.502, *P *= 0.002) (Figure 4B). However, no correlation was found with the SLICC damage index (ρ = 0.071, *P *= 0.658). There was no significant difference between the frequency of CD4^+^CD25^+^CD45RO^+ ^T cells in patients untreated or treated with corticosteroids (Kruskall-Wallis Test: z = 0.07, *P *= 0.94), antimalarial agents (Kruskall-Wallis Test: z = 1.41, *P *= 0.158), or both drugs (Kruskall-Wallis Test: z = -1.32, *P *= 0.132). Noteworthy, no correlation was found between total lymphocyte counts and the frequency of CD4^+^CD25^+^CD45RO^+ ^T cell (ρ = 0.021, *P *= 0.758), excluding the possibility that lymphopenia led to this phenotype. There was also no correlation with age (ρ = 0.153, *P *= 0.100).

### Variation of CD4^+^CD25^+^CD45RO^+ ^T cell frequency with the development and remission of SLE-related symptoms

**Figure 5:**
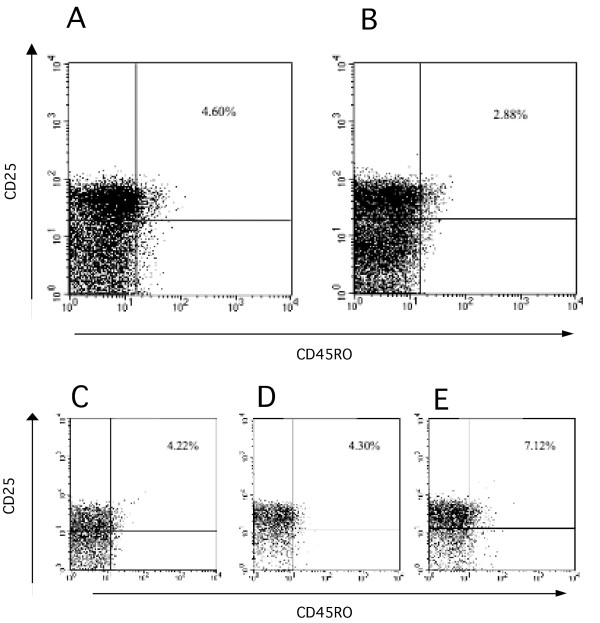
**CD4^+^CD25^+^CD45RO^+ ^T cell frequency of an SLE patient before (A) and after (B) SLE diagnosis**. (C) CD4+CD25+CD45RO+ T cell frequency of an SLE patient at the time of flare, (D) upon corticosteroid and anticoagulant therapy and (E) after IVIg administration. Lymphocytes gated according to forward and side scatter (FSC/SSC) from PBMC were screened by flow cytometry for the presence of CD4^+^CD25^+^CD45RO^+ ^T cells. The FACS panel representing CD4^+^CD25^+^CD45RO^+ ^is gated on CD4^+ ^cells.

In two SLE patients variations in CD4^+^CD25^+^CD45RO^+ ^T cell frequency as a function of disease onset and progression and with treatment were evaluated. A 33-year-old Caucasian woman was initially enrolled in this study as a healthy relative of an SLE patient. At the time of recruitment, the frequency of CD4^+^CD25^+^CD45RO^+ ^T cells in this subject was 4.60% (Figure 5A). One year later, this woman presented classical SLE clinical symptoms, lymphopenia, positive anti-dsDNA antibody, positive lupic anticoagulant and a SLEDAI of 10. Upon establishment of the diagnosis of SLE, a new measurement of Treg frequency showed a decrease to 2.88% (Figure 5B), suggesting the involvement of CD4^+^CD25^+^CD45RO^+ ^T cell frequency in the onset of symptoms. In a second case a 67-year-old female SLE patient, diagnosed three years before, developed an SLE flare with symptoms of acute isquemic stroke, hemolytic anemia, impaired renal function, antinuclear, anti-dsDNA and anticardiolipin antibodies and hipocomplementemia. The SLEDAI score was 42. A regimen of low-dose corticosteroids and antimalarials had previously provided a good control of disease. At this point the CD4^+^CD25^+^CD45RO^+ ^T cell frequency was 4.22% (Figure 5C). After anticoagulant and intravenous corticosteroid therapies, the CD4^+^CD25^+^CD45RO^+ ^T cell frequency was 4.30% (Figure 5D). In addition to anticoagulants and intravenous corticosteroids therapies, IVIg therapy administration was deemed necessary. Upon IVIg treatment, the patient's CD4^+^CD25^+^CD45RO^+ ^T cell frequency increased to 7.12% (Figure 5E) and she showed progressive clinical and laboratorial improvement, and the SLEDAI score decreased to 12 after three weeks of treatment. Taken together these results support the idea that decreased CD25^+ ^T cell frequency is associated with disease activity.

### Peripheral CD4^+^CD25^+^CD45RO^+ ^T cell frequency is highly heritable

Relatives of SLE patients presented intermediate CD4^+^CD25^+^CD45RO^+ ^T cell frequencies between patients and controls (Figure 1B), suggesting that Treg frequency might represent a genetic trait. Any potential bias due to recruitment design was excluded based on the following observations: for each family all samples were collected and processed on the same day; there were no significant differences in Treg frequency between families collected in different days. Treg frequencies in genetically unrelated family members and in controls collected and processed on the same day are within the range of healthy controls analyzed during the whole period of the study.

**Figure 6:**
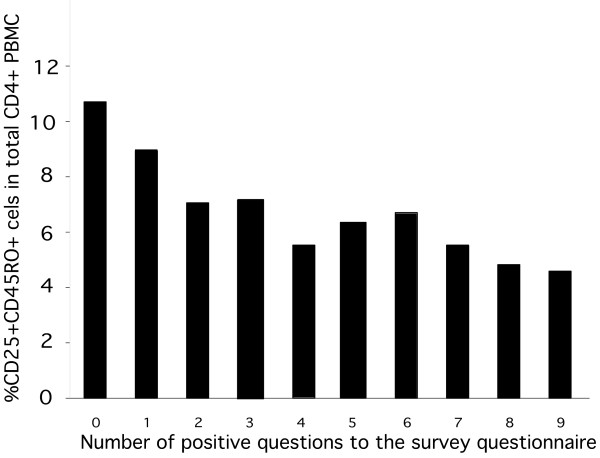
**Relative distribution of CD4^+^CD25^+^CD45RO^+ ^T cell frequency in patients and their relatives (n = 231) with the number of positive answers to the survey questionnaire, indicating a negative correlation between the frequency of CD4^+^CD25^+^CD45RO^+ ^T cells and the number of positive answers to the survey questionnaire**.

A high CD4^+^CD25^+^CD45RO^+ ^T cell frequency heritability, defined as the proportion of variation of this trait attributable to genetic factors, was estimated at 0.85 ± 0.09 (*P *= 1.05e-10) (Additional file 2). As for most complex traits, there was no clear segregation pattern of CD4^+^CD25^+^CD45RO^+ ^T cell frequency with affection status in the families. However, because relatives often present SLE-related autoimmunity symptoms even when not meeting the full diagnostic criteria, we tested the association of CD4^+^CD25^+^CD45RO^+ ^T cell frequency with the severity of these suboptimal autoimmune symptoms through a 10-item survey questionnaire administered to patients and relatives (Table 1). The number of positive answers was inversely correlated with the frequency of CD4^+^CD25^+^CD45RO^+ ^T cells (Kruskall-Wallis test: H = 20.7, *P *= 0.014) (Figure 6), as expected if the extent of clinical manifestations is associated with the frequency of CD4^+^CD25^+^CD45RO^+ ^T cells. The results therefore provide evidence that CD4^+^CD25^+^CD45RO^+ ^T cells frequency constitutes a genetic trait associated with SLE manifestations, which can be used for genetic mapping.

### Lower frequency of CD4^+^CD25^+^CD45RO^+ ^T cells is associated with specific *CTLA4 *and *TGFβ *gene variants

The evidence for familiar transmission prompted us to test putative functional polymorphisms in the candidate genes *CTLA4*, *TGFβ*, *FOXP3*, *IL2 *and *CD25 *for association with CD4^+^CD25^+^CD45RO^+ ^T cell frequency. All genotypic distributions were in HWE and all the polymorphic markers within the same gene were in LD. A significant association of the *CTLA4 *3'UTR microssatelite marker with CD4^+^CD25^+^CD45RO^+ ^T cell frequency was found (Kruskal-Wallis test: H = 40.57, *P *= 0.009), with genotypes 104/104 and 106/106 associated with lower (2.64%) and higher (5.47%) frequency of CD4^+^CD25^+^CD45RO^+ ^T cells, respectively (Table 2). This result is particularly relevant because, in our sample, allele 104 has previously been found to increase susceptibility to disease, while allele 106 showed a protective effect [27]. The *TGFβ *rs180047 SNP was also significantly associated with the frequency of CD4^+^CD25^+^CD45RO^+ ^T cells (Kruskal-Wallis test: H = 8.099, *P *= 0.004). Heterozygosis was associated with lower frequencies of CD4^+^CD25^+^CD45RO^+ ^T cells (Table 2). Because the C allele has very low frequency in the general population (6%), there were no homozygous individuals for this allele in our sample.

**Table 2:** Distribution of Treg frequency according to genotypes of the *CTLA4*, *TGFβ FOXP3*, *IL2 *and *CD25 *genes (non-parametric analysis of variance – Kruskall-Wallis test).

***CTLA4 *rs 231775 genotypes**	**N (%)**	**Median % CD4^+^CD25^+^CD45RO^+^Cells**	**Kruskall-Wallis Test**	***P***
A/A	39(13.22)	3.30		
A/G	115(38.98)	2.98	1.24	0.537
G/G	141 (47.80)	2.80		

***CTLA4 *3'UTR microssatelite genotypes**				

88/88	100 (38.02)	3.10		
88/104	9 (3.42)	2.66		
88/106	26 (10.27)	2.58	40.57	0.0009
104/104	3 (1.14)	2.54		
106/106	5 (1.90)	4.01		
Rare	119 (45.25)	2.81		

***TGFβ *rs18000469 genotypes**				

C/C	37(12.38)	3.55		
C/T	122 (40.80)	2.94	0.427	0.808
T/T	140 (46.82)	3.27		

***TGFβ *rs18000471 genotypes**				

G/G	255 (86.44)	3.29	8.10	0.004
G/C	41 (13.56)	2.10		

***FOXP3 *FOX0 microssatelite genotypes**				

1/4	3 (1.42)	2.54		
1/5	2 (0.95)	2.65		
4/4	68 (32.23)	3.15		
4/5	76 (36.02)	2.77		
4/6	21 (9.96)	2.58	9.06	0.911
4/7	3 (1.42)	5.22		
5/5	26 (12.32)	2.59		
5/6	6 (2.84)	2.08		
Rare	6 (2.84)	3.22		

***FOXP3 *FOX5 microssatelite genotypes**				

1/3	3 (1.59)	2.87		
1/4	4 (2.10)	3.79		
3/3	77 (40.74)	3.10		
3/8	5 (2.65)	2.54		
3/10	10 (5.29)	3.17		
3/12	13 (6.88)	2.99		
3/13	11 (5.82)	2.36		
3/14	43 (22.75)	2.51	9.06	0.911
3/15	3 (1.59)	3.16		
3/16	8 (4.23)	3.54		
3/18	3 (1.59)	2.74		
3/19	2 (1.06)	3.50		
7/13	2 (1.06)	3.76		
8/14	2 (1.06)	2.00		
Rare	3 (1.59)	3.19		

***IL2 *rs20699762 genotypes**				

G/G	137 (47.90)	3.55		
G/T	121 (42.31)	3.81	1.906	0.386
T/T	28 (9.79)	3.33		

***IL2 *rs3087209 genotypes**				

G/G	28 (9.82)	3.04		
G/T	101 (35.44)	3.81	0.944	0.624
T/T	156 (54.74)	3.16		

***CD25*D10S189 genotypes**				

1/1	7 (2.54)	2.58		
1/2	57 (20.73)	3.08		
1/3	1 (0.36)	4.06		
1/4	28 (10.18)	2.92		
1/5	21 (7.64)	3.37		
2/2	35 (12.73)	3.65	14.932	0.312
2/4	62 (22.54)	3.51		
2/5	32 (11.64)	3.25		
3/4	3 (1.09)	2.51		
4/4	11 (4.00)	2.68		
4/5	16 (5.82)	2.55		
5/5	2 (0.73)	5.77		

***CD25*rs2274037 genotypes**				

A/A	140 (83.83)	4.29	0.008	0.931
A/G	27 (16.17)	4.23		

***CD25*rs1570538 genotypes**				

C/C	50 (25.25)	4.43		
C/T	110 (55.56)	3.67	2.110	0.348
T/T	38 (19.19)	3.51		

N represents the number of individuals.

Both the *FOXP3 *rs12843496 SNP and the intron 0 marker, located in the promoter/enhancer region of the gene, were nominally associated with SLE (χ^2 ^= 6.14, *P *= 0.013 and χ^2 ^= 10.30, *P *= 0.036, respectively) (Table 3). The A allele of rs12843496 was more frequent in patients than in controls. The susceptibility allele 4 of the intron 0 microsatellite marker was more frequent in patients (χ^2 ^= 6.63, *P=*0.01) and a protective allele 6 more frequent in controls (χ^2 ^= 4.03, *P *= 0.04) (Table 3). No association was found with CD4^+^CD25^+^CD45RO^+ ^T cell frequency (Table 2). There was no evidence for association of any other tested gene marker with CD4^+^CD25^+^CD45RO^+ ^T cell frequency or with SLE (data not shown).

**Table 3:** Genetic association between *FOXP3 *markers and SLE.

	**Patients**	**Controls**			
**rs 12843496 allele**	**Number of alleles (%)**	**Number of alleles (%)**	χ^2^	***P***	**OR (95%CI)**
G	293 (96.7)	233 (100)			-
A	10 (3.3)	0 (0.0)	6.137	0.013	-
**Total**	303 (100)	233 (100)			

**FOX0 allele**					

1	7 (2.3)	4 (1.7)	0.04	0.84	1.38 (0.40–4.78)
4	189 (61.6)	121 (50.2)	6.63	0.01	1.59 (1.13–2.24)
5	97 (31.6)	93 (38.6)	2.61	0.10	0.74 (0.52–1.05)
6	11 (3.6)	19 (7.9)	4.03	0.04	0.24 (0.11–0.52)
Rare	3 (0.9)	4 (1.6)	0.10	0.75	0.81 (0.18–3.63)
**Total**	307 (100)	241 (100)	10.3	0.036	

**FOX5 allele**					

3	188 (63.7)	137 (57.9)	1.65	0.20	1.28 (0.90–1.82)
8	6 (2.0)	6 (2.6)	0.01	0.92	0.80 (0.25–2.49)
10	13 (4.4)	4 (1.7)	2.27	0.13	2.66 (0.86–8.28)
12	17 (5.8)	21 (8.9)	1.53	0.22	0.99 (0.54–1.80)
13	7 (2.4)	7 (3.0)	0.03	0.87	0.79 (0.27–2.29)
14	46 (15.6)	38 (16.2)	<0.01	0.95	0.96 (0.60–1.53)
15	3 (1.0)	5 (2.1)	0.43	0.51	0.47 (0.11–2.00)
16	7 (2.4)	9 (3.8)	0.52	0.47	0.61 (0.22–1.66)
18	0 (0.0)	5 (2.1)	4.26	0.04	-
Rare	8 (2.7)	4 (1.7)	0.23	0.63	1.61 (0.48–5.41)
**Total**	295 (100)	235 (100)	14.77	0.10	

**rs 12843496 genotypes**					

GG	136 (93.8)	77 (100)	3.51	0.06	-
GA	8 (5.5)	0 (0.0)	2.96	0.09	-
AA	1 (0.7)	0 (0.0)	0.11	0.74	-
**Total**	145 (100)	77 (100)	4.98	0.08	

**FOX0 genotypes**					

4/4	50 (33.8)	22 (25.3)	1.48	0.22	1.50 (0.83–2.72)
4/5	63 (42.6)	35 (40.2)	0.05	0.83	1.10 (0.64–1.88)
4/6	9 (6.1)	6 (6.9)	<0.01	0.98	0.87 (0.30–2.55)
5/5	12 (8.1)	13 (14.9)	2.02	0.16	0.50 (0.21–1.16)
Rare	14 (9.4)	11 (12.7)	0.30	0.50	0.72 (0.31–1.67)
**Total**	148 (100)	87 (100)	4.35	0.36	

**FOX5 genotypes**					

3/3	59 (33.8)	28 (33.3)	0.72	0.40	1.33 (0.76–2.32)
3/12	8 (5.4)	5 (6.0)	0.02	0.90	0.90 (0.29–2.85)
3/13	6 (4.1)	4 (4.8)	0.01	0.94	0.85 (0.23–3.08)
3/14	32 (21.6)	16 (19.1)	0.09	0.77	1.17 (0.60–2.29)
3/15	2 (1.4)	3 (3.6)	0.38	0.54	0.38 (0.06–2.32)
3/16	5 (3.4)	3 (3.6)	0.09	0.77	0.94 (0.22–4.05)
3/18	0 (0.0)	5 (6.0)	6.4	0.01	-
14/14	4 (2.7)	3 (3.6)	<0.01	0.98	0.75 (0.16–3.43)
Rare	32 (21.6)	17 (20.2)	0.01	0.94	1.09 (0.56–2.10)
**Total**	148 (100)	84 (100)	11.1	0.10	

Allele and genotype frequencies, χ^2 ^test and Odds Ratio for the *FOXP3 *rs12843496, intron 0 and intron 5 microssatelites in SLE patients and healthy individuals.

Taken together these results indicate that *CTLA4 *and *TGFβ *gene variants contribute to the determination of CD4^+^CD25^+^CD45RO^+ ^T cell frequency in PBMC, independently of disease activity, while the involvement of *FOXP3 *gene variants in SLE is likely not mediated by CD4^+^CD25^+^CD45RO^+ ^T cell frequency.

No correlation was found between CTLA-4, TGF-β, IL-2 or CD25 expression levels and the frequency of CD4^+^CD25^+^CD45RO^+ ^T cells, disease status or marker genotypes (data not shown). There was a trend towards higher levels of FOXP3 mRNA in SLE patients, although not significant (Mann-Whitney U-test: z = -1.78, *P *= 0.075), and in individuals carrying the A allele of *FOXP3 *rs12843496 (Mann-Whitney U-test: z = 4.95, *P *= 0.026). These results suggest that variability in CD4^+^CD25^+^CD45RO^+ ^T cell frequency associated with SLE is not influenced by altered expression of any of the tested genes.

## Discussion

The present study supports and expands previous observations of decreased CD4^+^CD25^+^CD45RO^+ ^T cell frequency in SLE patients. It further demonstrates a clear gender difference in the distribution of this regulatory T cell subset and provides evidence suggesting that CD4^+^CD25^+^CD45RO^+ ^T cell frequency variation may result from a defect in the conversion of FOXP3^+^CD25^- ^into FOXP3^+^CD25^+^cells. CD4^+^CD25^+^CD45RO^+ ^T cell frequency is shown to be negatively correlated with disease activity and with anti-dsDNA antibodies in the patient and control groups, and to change with development and remission of SLE symptoms in two documented cases. The study also established that the frequency of CD4^+^CD25^+^CD45RO^+ ^T cells is highly heritable in families affected with SLE, that it is negatively correlated with the severity of SLE-associated manifestations in patients and relatives, and that it is influenced by genetic variants in the *CTLA4 *and *TGFβ *genes.

We initially analyzed the frequency of Treg with the available anti-CD25 an CD45RO antibodies as described by Jonuleit et al. [35], who have demonstrated that human CD4^+^CD25^+^CD45RO^+ ^T cells are highly enriched in T cells with suppressor properties. CD25 and CD45RO are also markers of activation and one could argue that our measurement concerned conventional activated cells. Several observations indicate that this is not the case. First, the total frequency of CD45RO^+ ^cells was not significantly different between patients' and controls' PBMC, indicating that conventional activated cells are not enriched in SLE patients' PMBC. Second, once a FOXP3 antibody became available, we have tested this marker in a subset of patients. All together our analyses reveal that, when compared to controls, patients had a significantly decreased frequency of CD25^+ ^cells of either the CD4^+ ^CD45RO^+ ^or the FOXP3^+ ^phenotype, supporting the notion that the CD4^+^CD25^+^CD45RO^+ ^T cell subset is enriched in Treg. Hence, the analyses we presented here most likely concern *bona fide *Treg. Furthermore, while the CD4^+^FOXP3^+ ^cell subset was comparable between patients and controls, patients had a significantly higher frequency of FOXP3^+ ^CD25^- ^cells, suggesting that they are unable to convert FOXP3^+ ^CD25^- ^into FOXP3^+ ^CD25^+ ^cells. Previous observations endorse this hypothesis: SLE patients display multiple signaling defects leading to a defective IL-2 production [37], and CD4^+^FOXP3^+ ^thymocytes from IL-2^-/- ^mice have very few cells expressing CD25 when compared to wild type mice [38]. In fact, IL-2 does not seem to be required for the induction of FOXP3 expression but is required for the survival and expansion of Treg cells in the periphery, leading to autoimmune manifestations.

The percentage of CD45RO^-^CD31^+ ^RTE was not correlated with disease or with CD4^+^CD25^+^CD45RO^+ ^T cell frequency, ruling out a defect in thymic export. The faster decrease rate of CD4^+^CD25^+ ^CD31^+ ^cells in SLE patients, however, is indicative of a specific thymic defect leading to a decreased Treg production and thus contributing to the lower frequency of these cells in the periphery. The observed decrease of CD4^+^CD25^+^CD45RO^+ ^T cell frequency in patients might also result from cell migration to the sites of inflammation. However, this hypothesis seems unlikely because patients with rheumatoid arthritis display enriched CD4^+^CD25^+^CD45RO^+ ^T cells not only in inflamed joints but also in peripheral blood [39].

The difference found in CD4^+^CD25^+^CD45RO^+ ^T cell frequency between males and females is remarkable, given the much higher prevalence of SLE in females. This difference is very significant in the control population, suggesting this may be a gender specific trait that renders females more susceptible to SLE. The lack of significance in the patient group may be due to the very small number of males, while in the relatives' group this might be explained by the high heterogeneity of this population, where only some individuals carry the disease susceptibility factors.

The significant negative correlation of CD4^+^CD25^+^CD45RO^+ ^T cell frequency with anti-dsDNA antibodies observed in the patients and in the relatives suggests that these cells may be able to inhibit antinuclear antibody production by B cells, and therefore an additional mechanism through which loss of immunological tolerance may be achieved in SLE. This phenomenon may also occur in the relatives, but the fact that they display a positive correlation between CD4^+^CD25^+^CD45RO^+ ^T cell frequency and anti-dsDNA antibodies may represent a compensation effect in which the relatives' are able to overcome this pathogenic mechanism.

CD4^+^CD25^+^CD45RO^+ ^T cell frequency was also associated with disease activity and with the extent of clinical manifestations. This suggests a direct involvement in disease development and prognosis, which is corroborated by the variation observed with onset and remission of symptoms in the two documented cases. The frequency of CD4^+^CD25^+^CD45RO^+ ^T cells was not associated with the use of immunosuppressive drugs. Whether these therapies affect basal frequencies of Treg was not addressed in the present work as it would require longitudinal studies. Interestingly, in one case the clinical improvement that followed IVIg administration was associated with increased CD4^+^CD25^+^CD45RO^+ ^T cell frequency, suggesting that this treatment may influence Treg generation or maintenance [40].

The CD4^+^CD25^+^CD45RO^+ ^T cell frequency was highly heritable in the affected families and was also strongly correlated with the extent of autoimmune manifestations in patients and in affected and unaffected relatives, in concordance with the clustering of autoimmune diseases and/or autoimmune-related phenotypes observed in these families. These findings are of particular importance because they show a contribution of genetic factors to the decrease in CD4^+^CD25^+^CD45RO^+ ^T cell frequency and thus substantiate a genetic basis for SLE. Although we still cannot establish whether the decrease in CD4^+^CD25^+^CD45RO^+ ^T cells is a cause or a consequence of autoimmune manifestations in SLE, we show evidence for the existence of genetic factors that, by influencing this trait, may contribute to SLE.

The frequency of CD4^+^CD25^+^CD45RO^+ ^T cells likely results from a combination of processes regulating their expansion and survival in the periphery, and therefore multiple genetic factors may contribute to this trait. This study provided evidence for an involvement of the *CTLA4 *and *TGFβ *genes. The specific polymorphisms associated with CD4^+^CD25^+^CD45RO^+ ^cell frequency were not correlated with CTLA-4 and TGF-β mRNA expression level variation; expression levels in turn were not associated with cell variation or with disease status, indicating that a functional defect in these genes, rather than changes in expression levels, contributes to the observed CD4^+^CD25^+^CD45RO^+ ^T cell phenotype in SLE patients.

In mouse models the generation and expansion of Treg are dependent on CTLA-4 and TGF-β respectively, [41-46]. CTLA-4^-/- ^animals have reduced numbers of CD4^+^CD25^+ ^T cells with reduced regulatory capacity, while TGF-β regulates the Treg cell pool size and expression of the Treg commitment factor Foxp3 [11]. TGFβ has also been shown to induce CD4^+^CD25^+ ^regulatory T cells *in vitro*, which were able to prevent a murine lupus-like syndrome and graft versus host disease [47]. In humans, it was demonstrated that it has the capacity to expand CD4^+^CD25^+ ^in normal subjects [48]. Our results therefore extend the role of these genes in the regulation of Treg frequency and autoimmunity to humans.

Other Treg specific candidate genes that may contribute to the determination of the size of the Treg pool are the *FOXP3 *commitment factor and the *IL2 *and *CD25 *genes, which deliver crucial growth and/or survival signals to these cells. In the study population, a promoter polymorphism (rs12843496) in the *FOXP3 *gene was associated with SLE but not with the frequency of CD4^+^CD25^+^CD45RO^+ ^T cells, indicating that susceptibility to the disease is mediated by mechanisms other than influencing Treg frequency. The rs12843496 allele A associated with SLE creates a putative binding site to the transcriptional activator PLAG1 in the FOXP3 promoter region, which may explain the slightly higher FOXP3 mRNA expression found in SLE patients. A compensatory mechanism can be hypothesized to account for the maintenance of a normal mean CD4^+^FOXP3^+ ^cell frequency in patients even in the presence of increased frequency of this allele, and is plausible since FOXP3 can be upregulated by other cytokines.

The *IL2 *and *CD25 *genes were not associated with CD4^+^CD25^+^CD45RO^+ ^T cell frequency or with SLE. It should be remarked that the study population sample is small and that we focused on genotyping known functional polymorphisms because of their previously reported relevance, but did not fully cover genetic variation in any of these genes. Our genetic results should therefore be considered preliminary.

## Conclusion

In conclusion, we report that low peripheral CD4^+^CD25^+^CD45RO^+ ^T cell frequency is a genetically determined predisposing factor for SLE development. Our results demonstrate the contribution of specific genes and suggest pathways involved in the establishment of CD4^+^CD25^+^CD45RO^+ ^T cell frequency. Whether a defect in number or function is a prerequisite for SLE development remains to be understood. Most importantly, our findings open new perspectives for therapy for SLE patients based on the manipulation of Treg. Criteria for the selection of candidate patients for Treg based therapy remains to be fully established but likely should include evaluation of Treg frequency and analysis of genes involved in Treg generation or maintenance, strongly benefiting the management of this autoimmune disease.

## Authors' contributions

MB planed and conducted experiments, prepared the database, performed the statistical analyses and wrote the manuscript; RCF and LL conducted experiments; MFF and CC conducted experiments and performed clinical evaluation of patients and their relatives; ES and MA collected samples, performed clinical evaluation of patients and their relatives and prepared the database; BM, RA, and CV selected families for the study and supervised the blood collection; CF participated in the experimental design, selected families for the study, performed clinical evaluation of patients and their relatives and discussed the results; JFV participated in the autoantibody experiments; LMV collected samples and revised the manuscript; JD participated in the experimental design, discussed the results and wrote the manuscript; AV supervised the overall project, participated in the experimental design, discussed the results and wrote the manuscript. All authors read and approved the final manuscript.

## Supplementary Material

Additional file 1 **Total sample characteristics and samples used in each of the analyses.** Click here for file

Additional file 2 **Heritability test for CD4^+^CD25^+^CD45RO^+ ^regulatory T cell frequency in SLE families.** Click here for file

## References

[B1] Rocha B, von Boehmer H (1991). Peripheral selection of the T cell repertoire. Science.

[B2] Van Parijs L, Abbas AK (1998). Homeostasis and self-tolerance in the immune system: turning lymphocytes off. Science.

[B3] Itoh M, Takahashi T, Sakaguchi N, Kuniyasu Y, Shimizu J, Otsuka F, Sakaguchi S (1999). Thymus and autoimmunity: production of CD25+CD4+ naturally anergic and suppressive T cells as a key function of the thymus in maintaining immunologic self-tolerance. J Immunol.

[B4] Sakaguchi S (2004). Naturally Arising CD4+ Regulatory T Cells for Immunologic Self-Tolerance and Negative Control of Immune Responses. Annu Rev Immunol.

[B5] Hsu WT, Suen JL, Chiang BL (2006). The role of CD4CD25 T cells in autoantibody production in murine lupus. Clin Exp Immunol.

[B6] Bensinger SJ, Bandeira A, Jordan MS, Caton AJ, Laufer TM (2001). Major histocompatibility complex class II-positive cortical epithelium mediates the selection of CD4(+)25(+) immunoregulatory T cells. J Exp Med.

[B7] Waterhouse P, Penninger JM, Timms E, Wakeham A, Shahinian A, Lee KP, Thompson CB, Griesser H, Mak TW (1995). Lymphoproliferative disorders with early lethality in mice deficient in Ctla-4. Science.

[B8] Kwon H, Jun HS, Khil LY, Yoon JW (2004). Role of ctla-4 in the activation of single- and double-positive thymocytes. J Immunol.

[B9] Boden E, Tang Q, Bour-Jordan H, Bluestone JA (2003). The role of CD28 and CTLA4 in the function and homeostasis of CD4+CD25+ regulatory T cells. Novartis Found Symp.

[B10] Shull MM, Ormsby I, Kier AB, Pawlowski S, Diebold RJ, Yin M, Allen R, Sidman C, Proetzel G, Calvin D (1992). Targeted disruption of the mouse transforming growth factor-beta 1 gene results in multifocal inflammatory disease. Nature.

[B11] Peng Y, Laouar Y, Li MO, Green EA, Flavell RA (2004). TGF-beta regulates in vivo expansion of Foxp3-expressing CD4+CD25+ regulatory T cells responsible for protection against diabetes. Proc Natl Acad Sci USA.

[B12] Dieckmann D, Plottner H, Berchtold S, Berger T, Schuler G (2001). Ex vivo isolation and characterization of CD4(+)CD25(+) T cells with regulatory properties from human blood. J Exp Med.

[B13] Crispin JC, Martinez A, Alcocer-Varela J (2003). Quantification of regulatory T cells in patients with systemic lupus erythematosus. J Autoimmun.

[B14] Valencia X, Yarboro C, Illei G, Lipsky PE (2007). Deficient CD4+CD25high T regulatory cell function in patients with active systemic lupus erythematosus. J Immunol.

[B15] Kukreja A, Cost G, Marker J, Zhang C, Sun Z, Lin-Su K, Ten S, Sanz M, Exley M, Wilson B (2002). Multiple immuno-regulatory defects in type-1 diabetes. J Clin Invest.

[B16] Viglietta V, Baecher-Allan C, Weiner HL, Hafler DA (2004). Loss of functional suppression by CD4+CD25+ regulatory T cells in patients with multiple sclerosis. J Exp Med.

[B17] Hori S, Nomura T, Sakaguchi S (2003). Control of regulatory T cell development by the transcription factor Foxp3. Science.

[B18] Wildin RS, Smyk-Pearson S, Filipovich AH (2002). Clinical and molecular features of the immunodysregulation, polyendocrinopathy, enteropathy, X linked (IPEX) syndrome. J Med Genet.

[B19] Gavin M, Rudensky A (2003). Control of immune homeostasis by naturally arising regulatory CD4+ T cells. Curr Opin Immunol.

[B20] Kotzin BL (1996). Systemic lupus erythematosus. Cell.

[B21] Via CS, Handwerger BS (1993). B-cell and T-cell function in systemic lupus erythematosus. Curr Opin Rheumatol.

[B22] Dean GS, Tyrrell-Price J, Crawley E, Isenberg DA (2000). Cytokines and systemic lupus erythematosus. Ann Rheum Dis.

[B23] Hochberg MC (1997). Updating the American College of Rheumatology revised criteria for the classification of systemic lupus erythematosus. Arthritis Rheum.

[B24] Bombardier C, Gladman DD, Urowitz MB, Caron D, Chang CH (1992). Derivation of the SLEDAI. A disease activity index for lupus patients. The Committee on Prognosis Studies in SLE. Arthritis Rheum.

[B25] Johnson AE, Gordon C, Hobbs FD, Bacon PA (1996). Undiagnosed systemic lupus erythematosus in the community. Lancet.

[B26] Ferreira R, Barreto M, Santos E, Pereira C, Martins B, Andreia R, Crespo F, Viana JF, Vasconcelos C, Ferreira C (2005). Heritable factors shape natural human IgM reactivity to Ro60/SS-A and may predispose for SLE-asssociated IgG anti-Ro and anti-La autoantibody production. J Autoimmun.

[B27] Barreto M, Santos E, Ferreira R, Fesel C, Fontes MF, Pereira C, Martins B, Andreia R, Viana JF, Crespo F (2004). Evidence for CTLA4 as a susceptibility gene for systemic lupus erythematosus. Eur J Hum Genet.

[B28] Grainger DJ, Heathcote K, Chiano M, Snieder H, Kemp PR, Metcalfe JC, Carter ND, Spector TD (1999). Genetic control of the circulating concentration of transforming growth factor type beta1. Hum Mol Genet.

[B29] Tag CG, Mengsteab S, Hellerbrand C, Lammert F, Gressner AM, Weiskirchen R (2003). Analysis of the transforming growth factor-beta1 (TGF-beta1) codon 25 gene polymorphism by LightCycler-analysis in patients with chronic hepatitis C infection. Cytokine.

[B30] Bassuny WM, Ihara K, Sasaki Y, Kuromaru R, Kohno H, Matsuura N, Hara T (2003). A functional polymorphism in the promoter/enhancer region of the FOXP3/Scurfin gene associated with type 1 diabetes. Immunogenetics.

[B31] Fedetz M, Matesanz F, Caliz R, Ferrer MA, Collado MD, Alcina A, Martin J (2003). Lack of association between -384 and 114 IL-2 gene polymorphisms and rheumatoid arthritis. J Rheumatol.

[B32] Matesanz F, Caro-Maldonado A, Fedetz M, Fernandez O, Milne RL, Guerrero M, Delgado C, Alcina A (2007). IL2RA/CD25 polymorphisms contribute to multiple sclerosis susceptibility. J Neurol.

[B33] Almasy L, Blangero J (1998). Multipoint quantitative-trait linkage analysis in general pedigrees. Am J Hum Genet.

[B34] Abecasis GR, Cookson WO (2000). GOLD – graphical overview of linkage disequilibrium. Bioinformatics.

[B35] Jonuleit H, Schmitt E, Stassen M, Tuettenberg A, Knop J, Enk AH (2001). Identification and functional characterization of human CD4(+)CD25(+) T cells with regulatory properties isolated from peripheral blood. J Exp Med.

[B36] Kimmig S, Przybylski GK, Schmidt CA, Laurisch K, Mowes B, Radbruch A, Thiel A (2002). Two subsets of naive T helper cells with distinct T cell receptor excision circle content in human adult peripheral blood. J Exp Med.

[B37] Tenbrock K, Juang YT, Kyttaris VC, Tsokos GC (2007). Altered signal transduction in SLE T cells. Rheumatology (Oxford).

[B38] Fontenot JD, Rasmussen JP, Gavin MA, Rudensky AY (2005). A function for interleukin 2 in Foxp3-expressing regulatory T cells. Nat Immunol.

[B39] Cao D, Vollenhoven Rv R, Klareskog L, Trollmo C, Malmstrom V (2004). CD25brightCD4+ regulatory T cells are enriched in inflamed joints of patients with chronic rheumatic disease. Arthritis Res Ther.

[B40] Ephrem A, Chamat S, Miquel C, Fisson S, Mouthon L, Caligiuri G, Delignat S, Elluru S, Bayry J, Lacroix-Desmazes S (2008). Expansion of CD4+CD25+ regulatory T cells by intravenous immunoglobulin: a critical factor in controlling experimental autoimmune encephalomyelitis. Blood.

[B41] Read S, Malmstrom V, Powrie F (2000). Cytotoxic T lymphocyte-associated antigen 4 plays an essential role in the function of CD25(+)CD4(+) regulatory cells that control intestinal inflammation. J Exp Med.

[B42] Takahashi T, Tagami T, Yamazaki S, Uede T, Shimizu J, Sakaguchi N, Mak TW, Sakaguchi S (2000). Immunologic self-tolerance maintained by CD25(+)CD4(+) regulatory T cells constitutively expressing cytotoxic T lymphocyte-associated antigen 4. J Exp Med.

[B43] Manzotti CN, Tipping H, Perry LC, Mead KI, Blair PJ, Zheng Y, Sansom DM (2002). Inhibition of human T cell proliferation by CTLA-4 utilizes CD80 and requires CD25+ regulatory T cells. Eur J Immunol.

[B44] Annunziato F, Cosmi L, Liotta F, Lazzeri E, Manetti R, Vanini V, Romagnani P, Maggi E, Romagnani S (2002). Phenotype, localization, and mechanism of suppression of CD4(+)CD25(+) human thymocytes. J Exp Med.

[B45] Tang Q, Boden EK, Henriksen KJ, Bour-Jordan H, Bi M, Bluestone JA (2004). Distinct roles of CTLA-4 and TGF-beta in CD4(+)CD25(+) regulatory T cell function. Eur J Immunol.

[B46] Huber S, Schramm C, Lehr HA, Mann A, Schmitt S, Becker C, Protschka M, Galle PR, Neurath MF, Blessing M (2004). Cutting edge: TGF-beta signaling is required for the in vivo expansion and immunosuppressive capacity of regulatory CD4+CD25+ T cells. J Immunol.

[B47] Su H, Ye DQ, Wang BL, Fang XH, Chen J, Wang Q, Li WX, Zhang N (2008). Transforming growth factor-beta1-induced CD4+CD25+ regulatory T cells in vitro reverse and prevent a murine lupus-like syndrome of chronic graft-versus-host disease. Br J Dermatol.

[B48] Lu LY, Chu JJ, Lu PJ, Sung PK, Hsu CM, Tseng JC (2008). Expression of intracellular transforming growth factor-beta1 in CD4(+)CD25(+) cells in patients with systemic lupus erythematosus. J Microbiol Immunol Infect.

