# Supplementary material for: Low frequency of CD4+CD25+ Treg in SLE patients: a heritable trait associated with CTLA4 and TGFβ gene variants
Source: BMC Immunol. 2009 Jan 27;10:5. doi: 10.1186/1471-2172-10-5 (PMC2656467; doi:10.1186/1471-2172-10-5)
Supplement: Additional file 1 — Total sample characteristics and samples used in each of the analyses. [file 1471-2172-10-5-S1.doc]

**Additional File 1**

Additional file 1. Total study sample characteristics and samples used in each of the analyses.
