# Supplementary material for: Low frequency of CD4+CD25+ Treg in SLE patients: a heritable trait associated with CTLA4 and TGFβ gene variants
Source: BMC Immunol. 2009 Jan 27;10:5. doi: 10.1186/1471-2172-10-5 (PMC2656467; doi:10.1186/1471-2172-10-5)
Supplement: Additional file 2 — Heritability test for CD4+CD25+CD45RO+ regulatory T cell frequency in SLE families. [file 1471-2172-10-5-S2.doc]

**Additional file 2**

Additional file 2. Heritability test for the CD4^+^CD25^+^CD45RO^+^ regulatory T cell frequency in SLE families. In this test the likelihood of the sporadic model (null hypothesis) of no inheritance is compared with the likelihood of a polygenic model of inheritance (testing hypothesis). The models are then compared to find the hypothesis that is better supported by the data (χ^2^ = 2[lnLikelihood (polygenic model) – lnLikelihood (sporadic model)].

**Trait**

**%CD4+CD25+CD45RO+**

**LogLikelihood of the sporadic model**

**-104.68**

**Loglikelihood of the polygenic model**

**-84.49**

**Heritability**

**0.85**

**Chi-square**

**40.37**

***P***

**1.1e-10**
